# Next Generation Natural Killer Cells for Cancer Immunotherapy

**DOI:** 10.3389/fimmu.2022.886429

**Published:** 2022-06-02

**Authors:** Fiorella Rossi, Nathaniel Fredericks, Andrew Snowden, Michael J. Allegrezza, Uriel Y. Moreno-Nieves

**Affiliations:** Janssen Research and Development, LLC, Pharmaceutical Companies of Johnson & Johnson, Spring House, PA, United States

**Keywords:** NK cell, iPSC (induced pluripotent stem cell), CAR (chimeric antigen receptor), engineering, stealth

## Abstract

In recent years, immunotherapy for cancer has become mainstream with several products now authorized for therapeutic use in the clinic and are becoming the standard of care for some malignancies. Chimeric antigen receptor (CAR)-T cell therapies have demonstrated substantial efficacy for the treatment of hematological malignancies; however, they are complex and currently expensive to manufacture, and they can generate life-threatening adverse events such as cytokine release syndrome (CRS). The limitations of current CAR-T cells therapies have spurred an interest in alternative immunotherapy approaches with safer risk profiles and with less restrictive manufacturing constraints. Natural killer (NK) cells are a population of immune effector cells with potent anti-viral and anti-tumor activity; they have the capacity to swiftly recognize and kill cancer cells without the need of prior stimulation. Although NK cells are naturally equipped with cytotoxic potential, a growing body of evidence shows the added benefit of engineering them to better target tumor cells, persist longer in the host, and be fitter to resist the hostile tumor microenvironment (TME). NK-cell-based immunotherapies allow for the development of allogeneic off-the-shelf products, which have the potential to be less expensive and readily available for patients in need. In this review, we will focus on the advances in the development of engineering of NK cells for cancer immunotherapy. We will discuss the sourcing of NK cells, the technologies available to engineer NK cells, current clinical trials utilizing engineered NK cells, advances on the engineering of receptors adapted for NK cells, and stealth approaches to avoid recipient immune responses. We will conclude with comments regarding the next generation of NK cell products, i.e., armored NK cells with enhanced functionality, fitness, tumor-infiltration potential, and with the ability to overcome tumor heterogeneity and immune evasion.

## 1 Introduction

Cancer is a major public health problem worldwide and is the second leading cause of death in the United States, after cardiovascular disease (1, 2). Although a reduction in smoking and improvements in early detection and treatment have lowered the death rate of certain malignancies, such as non-small cell lung cancer (NSCLC), the overall survival of patients with late-stage malignancies remains poor—substantiating the need for novel therapeutic options. Treatment regimens for cancer have long consisted of surgery, radiation, and chemotherapy; but recently, a new approach has been on the rise: cancer immunotherapy.

Inspired by the natural capacity of our immune system to recognize and prevent tumor progression, *via* the concerted action of an array of immune cells and humoral factors, multiple immunotherapies have been developed (3). Early clinical studies of the therapeutic potential of stimulating T cells in cancer patients evidenced that administration of interleukin-2 (IL-2) can lead to durable tumor regressions in advanced cancers (4). Approval of IL-2 treatment for metastatic melanoma and renal cancer by the US Federal and Drug Administration (FDA) kickstarted a new era of cancer therapy and brought the immunotherapy in the spotlight (4, 5). The immuno-therapeutic arsenal for cancer continued to expand with the approval of immune checkpoint inhibitors (ICIs), which are directed against inhibitory molecules expressed on the surface of T cells or antigen-presenting cells (APCs) such as PD-1 and PD-L1, respectively, in several indications (6). More recently, chimeric antigen receptor (CAR)-T cells have shown therapeutic efficacy in treating refractory hematological malignancies, and several CAR-T cell therapy products have been approved by the FDA (7).

Despite their efficacy in treating cancer, current CAR-T cells therapies have limitations including life-threatening adverse events such as cytokine release syndrome (CRS) and neurotoxicity; additionally, CAR-T cell therapies are potentially susceptible to antigen escape of tumor cells and can cause detrimental on-target off-tumor effects (8). Additional limitations of current CAR-T cell therapy include complex and personalized manufacturing with time-sensitive and complex vein-to-vein logistics and the need of specialized centers for its application to patients.

Natural killer (NK) cells are a population of immune effector cells with potent anti-viral and anti-tumor activity and have recently emerged as a candidate for cancer immunotherapy. The biology of NK cells could potentially overcome some of the limitations of existing CAR-T cell products. For example, NK cells can recognize and kill tumor cells without the need of prior stimulation (9, 10). In addition, the response of NK cells is not major histocompatibility complex (MHC) restricted but relies on the ligation of multiple germline-encoded receptors, which enables CAR-NK cells to recognize and eliminate tumor cells even in the event of antigen loss or downregulation. Furthermore, multiple clinical trials have shown that NK cells are well tolerated and may have a safer profile than T-cell-based therapies (11).

In this review, we discuss advances on the development of engineered NK cells for cancer immunotherapy, including the sourcing of NK cells and the move towards off-the-shelf modalities, technologies to engineer NK cells, current clinical trials using engineered NK cells, advances on the engineering of receptors, and stealth approaches to avoid host immune response. In addition, we will comment on the next generation of NK cell products, i.e., armored NK cells with enhanced functionality, fitness, tumor-infiltration potential, and with the ability to overcome tumor heterogeneity and immune evasion.

## 2 Overview of NK Cells

### 2.1 NK Cell Populations

NK cells belong to group 1 innate lymphoid cells (ILCs) (12–15) and are characterized by their innate capacity to swiftly detect and kill aberrant cells, such as virus-infected cells and cancer cells (9, 10, 16). NK cells exhibit potent anti-tumor responses through multiple mechanisms and are functionally similar to cytotoxic CD8^+^ T cells.

Several populations of NK cells have been described. Most of our knowledge about human NK cells comes from peripheral blood NK (PBNK) cells, given that this population can be easily sampled. PBNK cells can be divided into two distinct subsets based on their expression of CD56 (also known as neural cell adhesion molecule 1): CD56^dim^ NK cells represent a mature population characterized by their innate cytotoxic potential, and CD56^bright^ NK cells represent a less mature population characterized by their immunomodulatory potential owing to their ability to secrete large amounts of cytokines upon stimulation (9, 16, 17). Additional populations of NK cells have been identified in multiple tissues where they play specialized immune functions. Tissue-resident NK cells have been observed in tissues such as the liver, gut, lungs, uterus, and kidney [reviewed elsewhere (18–21)].

Although NK cells belong to the innate immune system, certain populations of NK cells can display features attributed to the adaptive immune system such as antigen specificity and memory-like responses. Indeed, NK-cell populations mediating robust memory-like responses have been reported in the context of viral infections, contact hypersensitivity reactions, and after pro-inflammatory cytokine stimulation (22–25).

### 2.2 NK Cell Receptors and Target Cell Recognition

Unlike T cells, NK cells lack an antigen-specific T-cell receptor (TCR); instead, their activity is controlled by an array of germline-encoded receptors, both activating and inhibitory, that enable NK cells to sense their environment (26). The response of NK cells is modulated by the integration of signals delivered *via* activating and inhibitory receptors, and the balance of these signals determines the outcome of the interaction between NK cells and their environment, i.e., killing of target cells, secretion of cytokines, or no response.

Several activating receptors have been identified in NK cells; they recognize ligands expressed upon cell stress, viral infection, or tumor transformation. The major activating receptors involved in target cell recognition include NKG2D and natural cytotoxicity receptors (NCRs; namely, NKp46, NKp30, and NKp44). In addition, NK cells express the low-affinity Fc receptor CD16, which enables them to detect antibody-coated target cells and to exert antibody-dependent cell cytotoxicity (ADCC) (10, 26). NK cells are negatively regulated by inhibitory receptors, most of which ligate MHC class I molecules and gauge the level of expression of self-molecules on adjacent cells (10, 26–28). Human leukocyte antigen class I (HLA-I)–specific inhibitory receptors include the killer cell immunoglobulin-like receptors (KIRs) and the lectin-like CD94-NKG2A heterodimers. The interaction between self-MHC molecules and their cognate KIRs during NK cell development provides essential inhibitory signals for NK cell maturation and contributes to their education, i.e., their acquisition of functional competency (27, 28).

NK cells can kill target cells in a perforin-dependent manner, where following the formation of a lytic immunological synapse, preformed lytic granules containing perforin and granzymes converge toward the synapse and are released into the synaptic cleft (29). Perforin molecules form pores in the postsynaptic membrane of target cells allowing granzymes to enter the target cell and activate caspases, resulting in the apoptosis of the target cell (29–31). NK cells are protected from perforin-mediated autolysis by densely packed and highly ordered presynaptic lipid membranes (32). NK cells can also kill target cells in a perforin-independent manner, *via* the expression of FAS ligand (FasL) and tumor necrosis factor (TNF)-related apoptosis-inducing ligand (TRAIL) (9). Additionally, NK cells have immunomodulatory potential owing to their ability to secrete cytokines, chemokines, and growth factors, including interferon gamma (IFN-γ), TNF-α, CCL5, XCL1, and granulocyte-macrophage colony-stimulating factor (GM-CSF). As such, NK cells can positively or negatively influence the anti-tumor responses by modulating innate and adaptive immune cells (30, 33, 34).

### 2.3 NK Cells as Prognostic Value in Cancer

NK cells are key players of tumor immunosurveillance—they have the innate ability to differentiate healthy from malignant cells and mount an immune response following recognition of transformed cells. The clinical relevance of NK cells in cancer has been investigated, both in hematological and solid malignancies.

The abundance of NK cells has been correlated with prognosis in some hematological conditions, such as chronic lymphocytic leukemia (CLL), diffuse large B-cell lymphoma (DLBCL), T-cell lymphoma, and multiple myeloma (MM) (35). However, in acute lymphoid leukemia (ALL), the presence of NK cells in the bone marrow of children at the time of diagnosis was associated with favorable response to treatment and survival (36). A retrospective study of Hodgkin’s lymphoma (HL) patients showed that low numbers of infiltrating NK cells were associated with unfavorable clinical outcome (37). A prospective study of the relationship between the phenotype of NK cells at the time of ALL diagnosis and the minimal residual disease (MRD) at the end of induction chemotherapy showed that the presence of NK cells with a strong effector phenotype was associated with better leukemia control (38).

Additionally, evidence of the clinical relevance of NK cells in hematological malignancies comes from studies that linked mutations in genes essential for NK cell anti-tumor function with occurrence of cancer. As such, mutations of *PRF1* (encoding for perforin) are frequently found in patients with anaplastic large cell lymphoma (ALCL) and ALL, and mutation of *FASLG* (encoding for FasL) was observed in lymphoma patients (39–41). The secretion of IFN-γ by NK cells was found to be a positive prognostic marker in chronic myeloid leukemia (CML) (35), while reduced NK cell activity was observed in multiple malignancies, including advanced MM (42), and was associated with high-risk myelodysplastic syndrome (MDS) (43).

Concerning solid tumors, multiple studies in the early 2000s showed that increased infiltration of NK cells, based on CD57 expression, served as a positive prognosis factor in patients suffering from various malignancies. It was shown that increased infiltration of NK cells in squamous cell lung carcinoma, colorectal carcinoma, gastric carcinoma, and pulmonary adenocarcinoma correlated with better prognosis and survival of patients (44–47). In addition, in an 11-year follow-up prospective study of a cohort of Japanese general population, it was found that higher NK cell cytotoxicity was associated with reduced cancer risk, whereas low cytotoxicity was associated with increased cancer risk—in this cohort, the most frequent cancers identified were stomach, lung, and intestine (48).

More recently, a meta-analysis highlighted the important prognostic value of NK cell infiltration into a variety of solid tumors. High levels of NK cell markers in solid tumor tissues predicted favorable prognosis for solid tumor patients (49). Indeed, increased NK cell infiltration correlated with decreased risk of death; and in terms of localization, intraepithelial infiltration was more predictive of survival than NK cell infiltration into the tumor-adjacent stroma (50).

Cursons et al. showed that patients with metastatic melanoma have an improved survival rate if their tumor has a gene signature predicting NK cell infiltration, and high expression of IL-15 was associated with higher survival (51). IL-15 is an important cytokine for NK cell homeostasis and activation, and the presence of IL-15 within the TME was associated with NK cells with high anti-tumor function in head and neck squamous cell carcinoma (HNSCC) (52).

Additionally, NK cells were identified as a robust prognostic and predicative factor for chemotherapy outcome in gastric cancer (53). In metastatic melanoma patients undergoing ICI therapy, NK cell infiltration into tumor was correlated with favorable response to anti-PD-1 therapy, even in the event of tumor MHC-I downregulation (54).

Considering the association of NK cells with prognosis in several tumor malignancies and that NK cells can target tumor cells that have downregulated HLA-I molecules—an immune evasion mechanism often correlated with worse prognosis of cancer patients (55–57)—there is a window of opportunity to evaluate NK cell therapies, particularly in patients who have failed conventional immunotherapy due to tumors displaying reduced or non-existent HLA class I expression (58, 59).

## 3 NK cells for Cancer Immunotherapy

### 3.1 Early clinical studies using NK Cells

In the early 1980s, seminal studies by Rosenberg and colleagues showed that exposure of patient-derived peripheral blood mononuclear cells (PBMCs) to IL-2 alone generated cells that were able to recognize and kill tumor cells *in vitro* (4, 60, 61). These cells, which were named lymphokine-activated killer (LAK) cells, represented a heterogeneous mixture of T cells and NK cells. Phillips and Lanier later showed that a substantial part of the LAK anti-tumor activity was attributed to NK cells (62). Preclinical studies showed that adoptive transfer of LAK cells into tumor-bearing mice resulted in anti-tumor activity, and the concomitant administration of IL-2 with LAK cells increased *in vivo* anti-tumor activity of LAK cells (4, 63–65). These findings spurred an interest in this novel immunotherapy approach, resulting in clinical trials for advanced cancers using autologous LAK cells in combination with IL-2 infusions. However, in 1993, results from a randomized trial of 181 patients with advanced melanoma or renal cancer comparing treatment with IL-2 alone or in conjunction with LAK cells showed that the observed anti-tumor effects tended to be due to IL-2 alone (66), thereby hampering clinical studies of LAK cells.

Similarly, early clinical studies testing IL-2-activated autologous NK cells for treatment of patients with metastatic melanoma, renal cell carcinoma, relapsed lymphoma, and metastatic breast cancer were ineffective (67, 68), suggesting that in autologous settings, inhibitory signals from self-MHC molecules in tumor cells are likely to suppress NK cell function in the absence of activating signals. These investigators also noted a lack of persistence of the infused NK cells as a potential limitation.

In patients with acute myeloid leukemia (AML) undergoing hematopoietic stem cell transplant (HSCT), Ruggeri et al. showed a correlation between KIR profile and the outcome following HSCT (69), therefore suggesting that stratification of patients by their KIR ligand mismatch can select for patients with alloreactive NK cells that protect from AML relapse. Similar observations were made in additional studies, reporting decreased relapse and increased survival when patients received either HLA- or KIR-mismatched transplants (70–73).

In a landmark study in patients with poor-prognosis AML, Miller et al. found that the adoptive transfer of allogeneic IL-2-activated NK cells combined with lymphodepleting therapy resulted in a marked increase in endogenous IL-15, expansion of donor-derived NK cells, and induction of complete remissions in 26% of the patients (74). This study also highlighted the importance of lymphodepleting therapy in the creation of a cytokine milieu supportive of NK cell expansion. Using similar approaches, clinical responses were also observed when allogeneic NK cells were adoptively transferred into patients with refractory lymphoma and advanced MM (75, 76).

### 3.2 Clinical Trials of Engineered NK Cells

While the initial promising results from allogeneic NK cell adoptive transfer to cancer patients demonstrated the potential of NK cells, recent success of CAR-T cell therapies proved the value of targeting tumors with engineered receptors. These observations fused with the rapid progression of technologies available to engineer NK cells (**Table 1**) to set the stage for clinical trials of CAR-engineered NK cells in cancer (109).

**Table 1:** Technologies for the engineering of NK cells.

Technology	Description	References
**Non-viral delivery**		
**Electroporation**	Electroporation is one of the most used non-viral delivery strategies, resulting in high transfection efficiency of NK cells—particularly with mRNA. Electroporation has been used to generate functional CAR-NK cells, including CD19-, CD20-, and HER2-CAR NK cells with measurable increased cytotoxicity; and allows for co-transfection of CAR sequence with additional therapeutic nucleic acids. Among the disadvantages of electroporation, the risk of cytotoxicity and irreversible damage to the cell membrane due to high voltage, the transient expression of CAR, along with the unsuitability for large-scale manufacturing limit its clinical potential.	(77–81)
**Cell squeezing**	Cell squeezing is a microfluidic delivery approach in which cells are mechanically deformed as they pass through a constriction smaller than the cell diameter. The compression and shear forces result in the formation of transient holes that enable the diffusion of molecules into the cytosol. An advantages of cell squeezing is the possibility to co-transfect nucleic acids. Although the potential of this technology to engineer NK cells still needs to be further elucidated, Loo et al. recently reported that cell squeezing enables delivery of mRNA into primary NK cells with ~60% efficiency.	(82–84)
**Nanoparticles**	Multiple delivery approaches using nanoparticles have been developed, including lipid- and polymer-based. Nanoparticles are highly customizable with versatility for a variety of cargos, including transposons and CRISPR/Cas9 systems, and they can be designed for targeted delivery. For instances, polymer-based multifunctional nanoparticles with core-shell particles complexed with pDNA EGFR CAR can efficiently transfect NK cells and allow for monitoring of their trafficking *in vivo* through magnetic resonance and fluorescence optical imaging. Ionizable lipid nanoparticle (LNP) platforms allow stable formulation, endogenous cellular internalization, and low toxicity. Charge-altering releasable transporters (CARTs) efficiently transfect mRNA into primary human NK cells, including resting NK cells, with minimal impact on NK cell phenotype and function.	(85–87)
**Viral delivery**		
**Viral vectors**	Viral transduction allows for long-term and stable expression of transgenes—although it has an inherent risk of insertional mutagenesis. Both retroviral and lentiviral vectors have been used to engineer NK cells. Primary NK cells are resistant to transduction. To improve their transduction efficiency, NK cells can be pre-activated with cytokines or engineered K562 cells, follow multiple rounds of transduction, or incubated with reagents such as polybrene, DEAE-dextran, poly-L-lysine, fibronectin or retronectin. Additionally, pseudotyped vectors, such as Baboon envelope pseudotyped lentivirus, increase the affinity of the vector to NK cells resulting in higher transduction efficiency.	(88–93)
**Gene editing**		
**Transposons**	Transposons are “jumping” DNA elements that can change their position within the genome; the DNA transposon system involves a transposase that binds to terminal inverted repeats (TIRs) and mobilizes the DNA flanked by the TIRs. Transposons have low genotoxicity, cause less toxicity than viral transduction, and are suitable for co-delivery of multiple genes. The Sleeping Beauty (SB) DNA transposon system is capable of transposition in human cells and is currently used in several early clinical trials of CAR T cells. Using the SB system, Batchu, et al. generated mesothelin-CAR expressing NK-92 cells; and Bexte, et al. engineered primary NK cells with anti-CD19 CAR, with a safe genomic integration profile and high anti-tumor activity.	(85, 94–96)
**Designer nucleases**	Zinc finger nucleases (ZFN) and TALEN are the two most frequently used designer nucleases. The specificity of ZFN-mediated gene editing relies on its number of fingers, the amino acid sequence of the fingers, and the interaction of the nuclease domain. TALEN is composed of a DNA cleavage domain and a sequence-specific DNA-binding domain. Both ZFN and TALEN allow for specific editing with few off-target effects. TALEN have a simpler design than ZNF but are more difficult to deliver. The use of ZNF and TALEN have been limited in NK cells, in particular owing to the substantial protein engineering required for gene targeting.	(85, 97)
**CRISPR/Cas9**	The CRISPR/Cas9 system is composed of a programable single-stranded guide RNA (sgRNA) and a Cas9 endonuclease—mechanistically, the sgRNA binds to the target DNA sequence allowing the positioning of Cas9 at a specific site of the genome to make double-strand breaks, which can be followed by the integration of the desired gene cassette *via* endogenous DNA repair mechanisms. Advantages of CRISPR/Cas9 include its versatility to reach the target, and its potential for efficient and scalable manufacturing. However, CRISPR/Cas9 is less specific than ZNFs and TALENs and has a risk of off-target mutagenesis and immunogenicity. CRISPR/Cas9 system has been used to knockout *ADAM17* and *PDCD1* to improve NK cell functionality; also, it was used to develop *CISH^-/-^* iPSC-derived NK cells with improved metabolic fitness and enhanced functions. Additional CRISPR/Cas systems have been developed, such as CRISPR/Cas12a, increasing the options for CRISPR gene editing.	(98–100)
**Surface engineering**		
**Liposomes**	The functionality of NK cells can be enhanced by conjugating them with liposomes. Chandrasekaran et al. adorned liposomes with TRAIL and anti-NK1.1 *via* maleimide-thiol chemistry, allowing the ligation of the liposomes to NK cells. They showed that liposome-conjugated NK cells were retained in the tumor-draining lymph nodes, which resulted in the apoptosis of cancer cells and prevented metastasis. Also, NK cells can be engineered with drug-loaded nanoparticles. Siegler, et al. leveraging the tumor-specificity provided by CAR molecules, engineered CAR-NK cells with cross-linked multilamellar liposomal vesicles (cMLVs) containing the chemotherapeutic drug Paclitaxel (PTX). These CAR-NK cells adorned with PTX-loaded cMLVs showed enhanced antitumor efficacy in Her2- and CD19-overexpressing cancer models.	(101–103)
**Antibody-cell-conjugation (ACC)**	Antibody-cell-conjugation (ACC) technology enables the modification of cell surfaces with single-strand DNA (ssDNA). The modified cells are further annealed with the complementary strand-modified molecules. The ACC platform has been applied to link NK cells with transtuzumab (anti-HER2 mAb), allowing oNK cells (NK-92 cell line) to efficiently target HER2-expressing cancer cells *in vitro* and *in vivo*.	(104)
**Glycoengineering**	Glycoengineering allows the modification of the glycosylation of surface proteins to endow NK cells with new affinities and properties. This approach has been successfully used to the development of functional CD22-targeting NK-92 cells. Wang, et al. introduced high-affinity carbohydrate-based ligands for CD22 *via* metabolic engineering or glyco-polymer insertion. Hong, et al., used a chemoenzymatic glycocalyx editing strategy to introduce high-affinity and specific CD22 ligands onto NK cells and further functionalized NK cells with the E-selectin ligand sialyl Lewis X to increase infiltration into the tumor microenvironment.	(105, 106)
**Aptamers**	Aptamers are short single-stranded oligonucleotides often referred as “chemical antibodies” that can specifically recognize their targets, including nucleic acids and proteins, with high affinity in a similar manner to antibodies. Yang, et al. developed aptamer-engineered NK cells (ApEn-NK) with CD30-specific aptamers and showed that ApEn-NK were able to specifically target CD30+ T-cell lymphoma. Similarly, Chen, et al. developed ApEn-NK cells with PDGC21-T-specific aptamers; they showed the ApEn-NK cells were able to recognize triple-negative breast cancer (TNBC) cells and reduce lung metastasis *in vivo* in a TNBC xenograft model.	(107, 108)

Summary of the technologies available for delivery, gene editing and surface engineering of NK cells.

To induce tumor-specific responses, CAR-based therapies rely on the targeting of tumor antigens (TAs) by immune effector cells. Multiple CAR approaches have been tested in preclinical studies of CAR-NK cells that target antigens including CD19, EGFR, HER2, EpCAM, GD2, Mesothelin, and HSP70 [reviewed in (77)].

In 2020, Liu et al. reported results from a phase 1/2 trial using HLA-mismatched, cord-blood derived, anti-CD19 CAR-NK cells for the treatment of relapsed or refractory B-cell malignancies and showed that 73% of patients responded, with seven of eight patients in complete remissions following therapy (110). They also reported that administration of CAR-NK cells was not associated with CRS, neurotoxicity, nor graft-versus-host disease (GvHD), therefore highlighting a safety profile of CAR-NK cell therapy.

The number of clinical trials exploring the potential of engineered NK cells for cancer therapy has steadily increased over time. In the current landscape, there are over 20 ongoing clinical trials using CAR-NK cells for hematological and solid indications (**Table 2**). One attractive CAR-NK cell strategy is the use of multiplexed induced pluripotent stem cells (iPSC)-derived NK (iNK) cells engineered to express CARs alongside additional edits, including IL-15 receptor fusion and high-affinity CD16 (114). Multiple clinical trials are evaluating iNK cells in hematological malignancies, such as MM, AML, CCL, and B-cell lymphoma, in combination with antibody therapy (daratumumab, elotuzumab, rituximab, and obinutuzumab, respectively), and iNK cells are being trialed in solid tumors, including ovarian cancer (**Table 2**).

**Table 2:** List of ongoing clinical trials utilizing engineered NK cells.

TumorType	Target(s)	Disease Condition	Source of NK cells	NK cell drug candidate	Combination biological agent	Company/Sponsor	Phase	Reference
**Hematologic Malignancies**	CD19	ALL, CLL, NHL	Cord blood	iC9/CAR.19/IL15 CB-NK cells	–	M.D. Anderson Cancer Center	Phase 1/2	NCT03056339
	CD33	AML	Unknown	Anti-CD33 CAR-NK cells	–	Sichuan Kelun-Biotech Biopharmaceutical Co., Ltd.	Phase 1	NCT05008575
	NKG2D ligands	AML, MDS	Peripheral blood, allogeneic	NKX101	–	Nkarta Inc.	Phase 1	NCT04623944
	CD19	NHL	Unknown, Allogeneic	CAR-NK019	–	Zhejiang University	Phase 1	NCT04887012
	BCMA	MM	Cord blood	Anti-BCMA CAR-NK cells	–	Sichuan Kelun-Biotech Biopharmaceutical Co., Ltd.	Early Phase 1	NCT05008536
	CD33 + CLL1	AML	Unknown	Anti-CD33/CLL1 CAR-NK cells	–	Imbioray (Hangzhou) Biomedicine Co., Ltd.	Early Phase 1	NCT05215015
	CD19	ALL, CLL, NHL	Cord blood	Anti-CD19 CAR-NK cells	–	Shanghai Simnova Biotechnology Co.,Ltd.	Phase 1	NCT04796675
	BCMA	MM	NK-92 cell line	Anti-BCMA CAR-NK-92 cells	–	Asclepius Technology Company Group (Suzhou) Co., Ltd.	Phase 1/2	NCT03940833
	CD19	Leukemia, Lymphoma	Peripheral blood, allogeneic	NKX019	–	Nkarta Inc.	Phase 1	NCT05020678
	BCMA + CD38	MM	iPSC	FT576	Daratumumab (anti-CD38 mAb)	Fate Therapeutics, Inc.	Phase 1	NCT05182073, (111)
	SLAMF7 or CD38	AML, MM	iPSC	FT538	Daratumumab (anti-CD38 mAb), Elotuzumab (anti-SLAMF7 mAb)	Fate Therapeutics, Inc.	Phase 1	NCT04614636
	CD19 + CD20	B-cell lymphoma, CLL	iPSC	FT596	Rituximab (anti-CD20 mAb), Obinutuzumab (anti-CD20 mAb)	Fate Therapeutics, Inc.	Phase 1	NCT04245722
**Solid Tumors**	NKG2D ligands	Metastatic CRC	Unknown	NKG2D CAR-NK cells	–	Zhejiang University	Phase 1	NCT05213195
	HER-2	GC, MBC	NK-92 cell line	ACE1702	–	Acepodia Biotech Inc.	Phase 1	NCT04319757, (104, 112)
	B7-H3	OC, FTA, PPC	iPSC	FT516	Enoblituzumab (anti-B7-H3 mAb), IL-2	Masonic Cancer Center, University of Minnesota	Phase 1	NCT04630769
	PD1 ligands	NSCLC	NK-92 cell line	CCCR-NK-92 cells	–	Xinxiang medical university	Phase 1	NCT03656705, (113)
	PD-L1	Solid tumors	iPSC	FT516	Avelumab (anti-PD-L1 mAb), IL-2	Fate Therapeutics, Inc.	Phase 1	NCT04551885
	HER-2	GBM	NK-92 cell line	NK-92/5.28.z	–	German Cancer Research Center	Phase 1	NCT03383978
	5T4	Solid tumors	Unknown, Allogeneic	Anti-5T4 CAR-raNK cells	–	Shanghai East Hospital	Early Phase 1	NCT05137275
	PD-L1	Pancreatic Cancer	NK-92 cell line	PD-L1 t-haNK	N-803	ImmunityBio, Inc.	Phase 2	NCT04390399
	ROBO1	Solid tumors	Unknown	ROBO1 CAR-NK cells	–	Asclepius Technology Company Group (Suzhou) Co., Ltd.	Phase 1/2	NCT03940820
	PD-L1 + PD1	GEJ, HNSCC	NK-92 cell line	PD-L1 t-haNK	Pembrolizumab (anti-PD1 mAb), N-803	National Cancer Institute	Phase 2	NCT04847466

List of active clinical trials (recruiting, enrolling by invitation, active not recruiting) involving engineered NK cells, obtained from https://clinicaltrials.gov/ on February 8, 2022.

ALL, acute lymphocytic leukemia; AML, acute myeloid leukemia; CLL, chronic lymphocytic leukemia; CRC, colorectal cancer; FTA, fallopian tube adenocarcinoma; GBM, glioblastoma; GC: gastric cancer; GEJ, gastroesophageal junction cancer; HNSCC, head and neck squamous cell carcinoma; MBC, metastatic breast cancer; MDS, myelodysplastic syndromes; MM, multiple myeloma; NHL, non-Hodgkin lymphoma; NSCLC, non-small cell lung cancer; OC, ovarian cancer; PPC, primary peritoneal cavity cancer.

Overall, clinical trials have demonstrated that NK cells possess potent anti-tumor effects without eliciting serious adverse effects associated with T-cell therapy, such as GvHD (115, 116), neurotoxicity (117), or cytokine release syndrome (118).

### 3.3 Current Sources of NK Cells for Therapy

**Figure 1:**
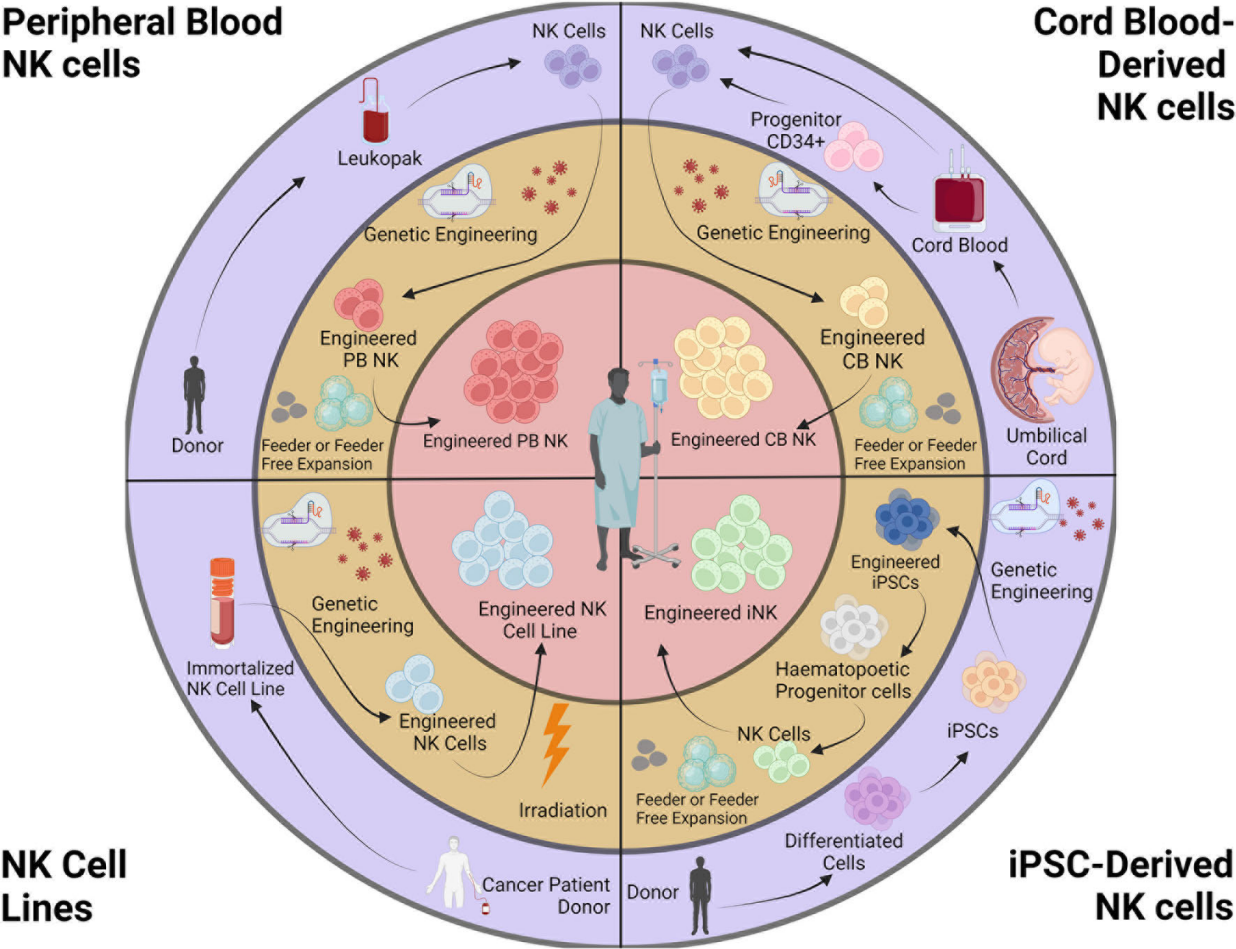
Sources and engineering of NK cell products. NK cells for cancer immunotherapy can be obtained from peripheral blood (autologous or allogeneic), cord blood, iPSCs, and NK cell lines. Isolated NK cells are genetically engineered and expanded. NK cell lines are irradiated before infusion. This figure was created using BioRender.

CAR-expressing NK cells are derived from a variety of sources, including peripheral blood (PB), umbilical cord blood (CB), iPSCs, and NK cell lines (**Figure 1**). Recent reviews have described the sources of NK cells in detail, with their advantages and caveats (77, 88).

Whereas obtaining a suitable source of autologous PBNKs is a challenge because patients typically have received prior therapies, CBNKs typically exhibit a naive phenotype including lower expression of adhesion molecules (e.g., CD2, CD11a, CD18, and CD62L), CD16, KIRs, perforin, and granzyme B, resulting in decreased cytotoxicity (119, 120). NK cells can also be derived from cord-blood hematopoietic progenitor CD34^+^ cells (121, 122). However, these sources have the limitation of poor product standardization due to the heterogeneous nature of PB and CBNKs. Genetically modifying these primary NK cells remains highly variable using currently available technologies, resulting in difficulties developing consistent and reproducible engineered NK cells (123). Although the cancer-derived NK cell line NK-92 can overcome the challenges above, the obvious safety requirement for being mitotically inactivated by irradiation creates significant limitations to potential clinical use with patients (124). Without the ability for proliferation upon infusion, their anti-tumor activity is rapidly reduced overtime when compared to alternative NK cell therapies (74, 125). Indeed, a phase I study with NK-92 cells transduced with a CAR targeting CD33 were tested in patients with relapsed or refractory AML but showed no durable responses partly due to lower persistence and efficacy due to irradiation prior to treatment (126).

**Figure 2:**
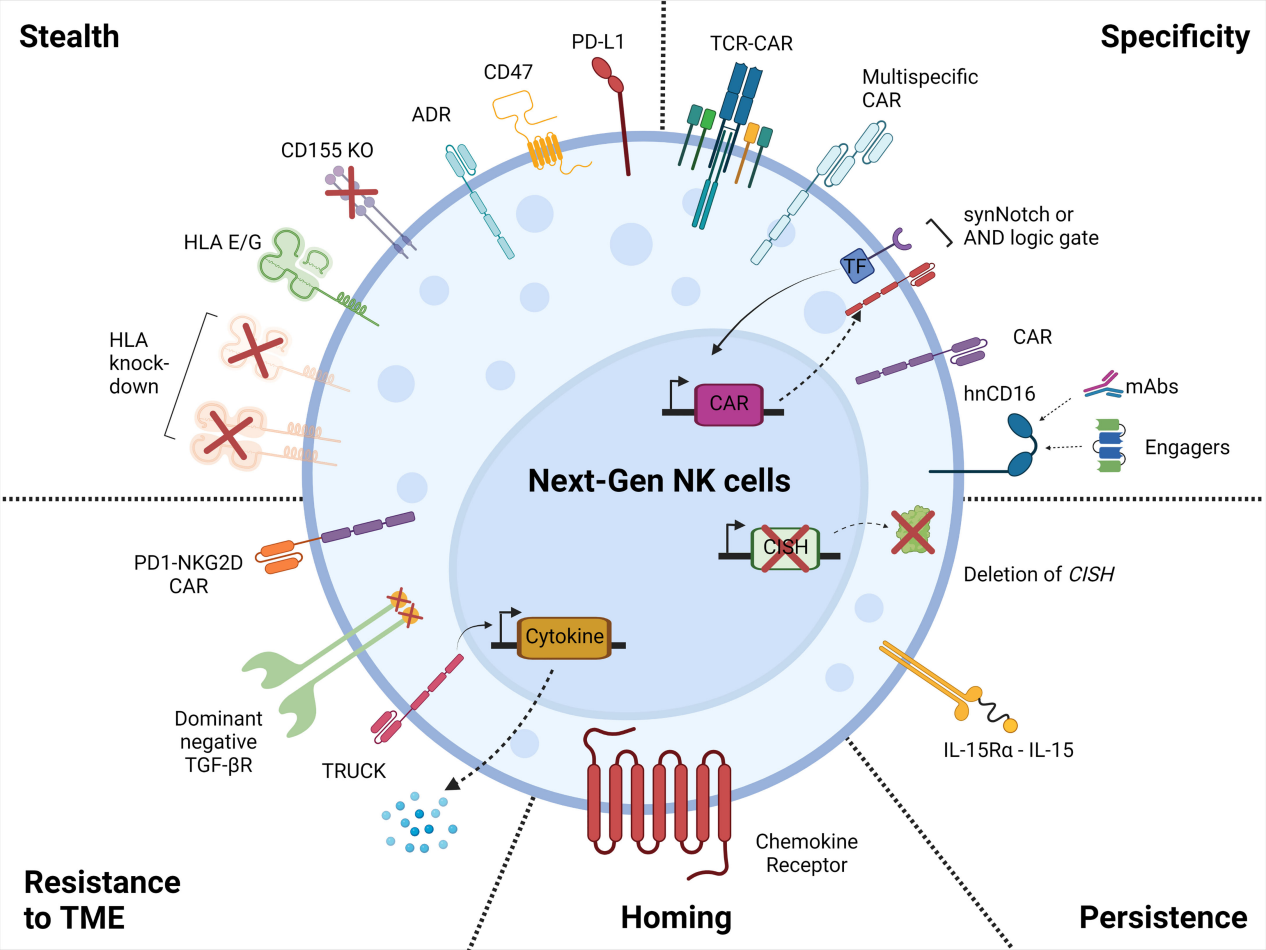
Next generation NK cell products. Illustration of the attributes of the next-generation NK cell products with increased tumor-targeting specificity, persistence, homing, resistance to the tumor microenvironment, and with stealth capabilities. This figure was created using BioRender.

On the contrary, human iPSCs are a source for cell therapy that can be genetically engineered *via* established methods, expanded and produced indefinitely in a homogenous and limitless manner (127, 128), and can be differentiated into iNK cells (129). iPSC-derived NK cells have inherent safety considerations; indeed, given that iPSC can proliferate indefinitely, careful analysis of the final iNK product needs to be undertaken to ensure that it is free from residual iPSCs (130). These benefits can be translated into a highly versatile and standardized off-the-shelf iNK cell therapy to treat various malignancies (110, 131), with the possibility to make multiple precision edits at the single-cell level to produce banks of homogeneous NK-cell products with improved persistence, tumor targeting, homing, and functionality (132) (**Figure 2**).

## 4 Developing the Next Generation of NK Products

### 4.1 Engineered Receptors for Targeting Tumors

Engineering CARs onto an immune cell redirects their specificity onto a particular antigen. The first proof of concept for antigen specificity stemmed from combining the signaling of a T cell with the antigen specificity of an antibody *via* fusing the variable regions of an antibody with the constant region of TCR (133, 134). This CAR technology was developed further by utilizing the commonly utilized antigen-binding domain known as single-chain variable fragment (scFv; containing the variable heavy and light chains from an antibody) leading to “first-generation” CAR constructs. These have proven to be capable of eliciting tumor-specific cytotoxicity, as demonstrated by human epidermal growth factor receptor 2 (HER2)-specific CARs using scFv recognizing HER2 fused to a CD3ζ signaling domain for activation (signal 1) (135, 136). However, these first-generation CARs failed to elicit significant anti-tumor responses due to limited persistence (137) and expansion *in vivo* (138).

Endogenous TCR cellular signaling requires both the TCR together with costimulatory (signal 2) or accessory molecules to elicit a robust response, which can be adapted into a single CAR molecule *via* molecular engineering. By taking advantage of the modular nature of CAR receptor technology, this allowed for the continual refinement and modification of these engineered proteins to optimize functionality, leading to improved second- and third-generation CARs containing one or two costimulatory domains, respectively. Despite promising results for various hematological malignancies (110, 139–143), the therapeutic efficacy of CAR-T approaches was limited in both hematological (143, 144) and solid tumors (145, 146). These limitations have been related to diminished cytotoxicity, inefficient trafficking, and infiltration of tumors in an immunosuppressive environment where heterogeneity in tumor target expression can often occur. Initiatives to overcome these shortcomings have led to various strategies including engineering fourth-generation CARs to produce and release a transgenic product, such as pro-inflammatory cytokine IL-12, which is constitutively or inducibly expressed upon CAR activation (147, 148). T cells that are transduced with these fourth-generation CARs are generally referred to as T cells redirected for universal cytokine-mediated killing (TRUCKs) and will be further discussed in later sections.

The development of next-generation NK cells for cancer therapy has been facilitated by the emergence of sophisticated technologies enabling the engineering of NK cells (**Table 1**) in terms of delivery (viral vectors, electroporation, cell squeezing, and nanoparticles), gene editing (transposons, designer nucleases, and CRISPR/Cas systems), and surface engineering (liposomes, antibody–cell conjugation, glycoengineering, and aptamers).

### 4.2 “Armoring” of NK Cells Through Enhanced Functionality

Engineering genetic modifications to augment persistence and functionality can be easily performed using a single-cell iPSC engineering platform. This was demonstrated in iNK cells with the addition of a modified high-affinity version of CD16a (149). NK cells naturally express CD16a, which binds the Fc portion of immunoglobulin G (IgG) attached to target cells and induces ADCC. Engagement of CD16a alone is sufficient for inducing NK cell activation, leading to secretion of various inflammatory cytokines and chemokines for the recruitment and activation of other immune cells (150–152). However, CD16a undergoes rapid proteolytic cleavage upon stimulation mediated by ADAM17 (153–155). Substitution of serine at position 197 located in the middle of the cleavage region with proline (S197P) effectively prevents cleavage of the intact and functional receptor (156). In addition, the binding affinity of CD16a varies between allelic variants (157). This led to the discovery of a high-affinity CD16a variant with valine at position 158 (158V) (158); patients with this variant had greater objective responses and progression-free survival when treated with mAb therapy such as rituximab (159), cetuximab (160), or trastuzumab (161). Engineered iNK cells with a high-affinity non-cleavable version of CD16a (hnCD16) displayed enhanced ADCC effector function when combined with anti-tumor mAbs (131).

NK cells do not naturally produce signal 3 cytokines such as IL-2, IL-7, or IL-15, which is in contrast to polyclonal CAR-T cell therapies that, upon CAR engagement, produce significant amounts of IL-2 that supports their expansion (162). Because NK cells rely on signal 3 for expansion, survival, and cytotoxicity, investigators have focused on ways to provide this signal to NK cell therapies, either exogenously or by design in the NK cell product. Optimizing CAR signaling with the addition of signal 3, which includes the transgenic expression of cytokines or a chimeric cytokine receptor, further enhances cytotoxicity and persistence of NK cells. The TME is often deficient in signal 3, thus hampering the potential degree of anti-tumor response of cell therapies. As previously mentioned, fourth-generation TRUCK CAR-T cells have been engineered to minimize systemic toxicity and to induce targeted accumulation of cytokines at the tumor site by providing signal 3. To enhance both NK durability and host immune responses, initial clinical trials focused on the administration of cytokines such as IL-2 and IL-15 for safety and efficacy. However, IL-2 administration resulted in severe systemic toxicity (163), which prompted the focus of investigations onto IL-15. In contrast to IL-2, treatment with IL-15 did not promote expansion of inhibitory Tregs (164) and activation-induced cell death (AICD) of T cells (165). Clinical trials combining NK cell therapy and IL-15 cytokine support resulted in limited anti-tumor responses in patients due to a short half-life after delivery (166, 167). To enhance and maintain the therapeutic effectiveness of IL-15, a membrane-bound IL-15/IL-15 receptor fusion (IL-15RF) was generated, resulting in increased persistence, proliferation, and enhanced cytotoxicity in iNK cells (131, 168). Deletion of cytokine-inducible SH2-containing protein (CIS, encoded by *CISH*), a negative regulator of IL-15 signaling, further improved cytotoxic effector function, metabolic fitness, and *in vivo* persistence of iNK cells (98, 169).

### 4.3 Strategies to Overcome Tumor Heterogeneity and Immune Evasion

Heterogeneous antigen expression in tumor cells led to the development of CAR-T cells targeting simultaneously two or more tumor-associated antigens either by a tandem (170–172) or split CAR construct configuration (173). Indeed, both anti-CD19 and anti-CD22 CAR-T cell therapies have shown impressive efficacy; however, patients have shown reduced antigen density at relapse, suggesting tumor-antigen-specific downregulation as a mechanism for immune escape (140, 174). Currently, the optimal strategy for multi-targeting is being investigated between tandem or separate CAR constructs in T cells regarding safety and efficacy of these constructs (170, 172, 175). However, having a heterogeneous mixed product resulting from transducing T cells with either a tandem or bicistronic CAR has proven to be a complex manufacturing procedure and makes mechanistic investigations difficult. This could potentially be overcome by utilizing a renewable source for consistent and selective gene editing (131). In another example, targeting B-cell maturation antigen (BCMA) has shown extremely promising and potentially durable objective response rates in MM (139). However, gamma-secretase (GS)-mediated cleavage of BCMA releases soluble BCMA fragments that have been shown to be capable of inhibiting BCMA-CAR function, leading to the testing of combinatorial therapy with GS inhibitors to prevent antigen escape (176). Targeting pan-cancer antigens such as CD276 (B7-H3) has also shown tremendous success in treating various solid malignancies; however, their success is largely dependent on the level of surface antigen density (177). Although multi-targeting or combinatorial cleavage inhibition may address tumor immune evasion through antigen loss, it does not address other resistance mechanisms such as inhibition by engagement of PD-L1 on tumor cells (178) or navigating through the immunosuppressive TME.

While these multi-specific CAR strategies are developed to target tumor-associated antigens—which may be also expressed in healthy tissues—they could result in severe on-target off-tumor toxicities similar to those seen in specific cases with some CAR-T cell therapy trials (179–181). Strategies to circumvent this issue have led to engineering approaches to insert logic gates into the cells, resulting in activation in the presence of combinations of target antigens. Utilizing a split CAR model by separating signal 1 and signal 2 into different CARs, each with a binder for a different antigen, has now led to the development of an AND logic gate (182). Although intracellular CD3ζ domain for signal 1 by itself is sufficient for signaling, Kloss et al. modified the CD3ζ domain to make it insufficient to produce an activating signal without the co-stimulation by a secondary CAR with signal 2 (183). Another strategy of utilizing AND logic gating is by inducing expression of the primary tumor-targeting CAR only in response to a secondary antigen such as the SynNotch system (184). To overcome the potential off-target recognition of healthy cells, inhibitory NOT logic gates can be engineered to ignore cells that express a particular antigen that is expressed in healthy tissue but not in tumors. The NOT logic CAR was initially conceived by fusing the intracellular domain of PD1 or CTLA4 with a targeting domain recognizing antigens expressed on healthy tissues, thus allowing the primary tumor-targeting CAR to selectively kill tumor cells (185). Naturally, for any of these approaches to work requires identification of combinatorial expression patterns that are truly unique to tumors. In addition, a distinct separation between healthy and tumor cells must be present; otherwise, unintended activation or inactivation may still potentially occur. The recent improvements in engineering multiple edits into cell therapy products demonstrate the benefits of logic circuitry if the appropriate antigen combinations can be identified.

Beyond the use of exquisite, but synthetic, logic circuitry for efficient tumor targeting comes augmenting cells with CARs based on natural cytotoxicity ligands native to NK cells to target heterogeneous tumors. Several groups have demonstrated the use of NKG2D-based CAR therapy with unique intracellular signaling domains, such as a second-generation CD3ζ/CD28 (186) or NK-inspired DAP12 (187), to target various tumor and immunosuppressive cells. Alternatively, NKp46 (188), NKp44 (189), and NKp30 (190) have each been fused to generate second-generation CAR-T cells that further demonstrates the benefit of tumor recognition capability of natural NK ligands for anti-tumor efficacy. This concept was taken further by utilizing an scFv targeting B7H6, a ligand for NKp30, to create a CAR that targets multiple tumor cells while demonstrating an impressive safety profile, since B7H6 is not constitutively expressed on healthy tissues (191). Indeed, utilizing NK ligands for the recognition of heterogeneous tumor targets provides a selective but effective way to overcome evasion by tumor cells to T-cell therapies. Alternatively, NK-insensitive cancer cell lines that are non-targetable by NKG2D-mediated cytotoxicity have resulted in the generation of NK cells expressing a TCR-CAR (192). This allows the TCR-CAR to redirect cytotoxic effector cells such as NK-92 cells by endowing them the ability to recognize tumor cells typically elusive to NK cell detection. Indeed, expressing the TCR in NK cells could greatly enhance the range of targetable antigens by enabling recognition of tumorigenic neoantigens within the intracellular proteome. However, this strategy can potentially give rise to TCR-mediated GvHD. There is also a limitation due to the diversity of MHC alleles and the likelihood that a particular TCR will only recognize a neoantigen when it is presented by a specific MHC allele. Thus, TCRs would have to be selected to recognize a neoantigen presented in a wide range of MHC alleles to minimize GvHD. One potential clone, MC.7.G5, appeared to recognize a currently unknown cancer-specific molecule presented by the non-polymorphic protein MHC-related 1 (MR1) (193). Since MR1 is a member of a family of non-classical MHC proteins, this TCR should enable recognition of cancer cells in a wide range of patients while minimizing off-target effects. Indeed, further investigation will be required for TCR-CARs to assess whether their off-target potential is worth their clinical impact.

### 4.4 Targeting Negative Regulators

Successful treatment of solid tumors has been elusive in part due to the immunosuppressive nature of the TME. Tumor cells can evade immune surveillance by secreting or promoting the secretion of transforming growth factor beta (TGF-β) (194). Because of its suppressive role in the TME, TGF-β has been targeted to boost cell therapy anti-tumor response. TGF-β mediates downregulation of NKG2D, NKp30, TRAIL, and DNAM1 receptors on activated NK cells (195, 196). To shield adoptive NK cell therapies from the suppressive effects of TGF-β, introduction of a dominant negative form of TGF-β type II receptor (TGF-βRII) efficiently blocked TGF-β signaling and maintained cell surface expression of receptors and cytotoxicity in NK and T cells (197–199). Elegant strategies embracing the inhibitory cytokine and converting it into a potent stimulatory signaling have been created by rewiring the recognition domain into a second-generation CAR-T cell to orchestrate upregulation of cytokine production and proliferation (200). Similarly, expressing a CAR with a TGF-βRII extracellular and transmembrane domains combined with the intracellular domain of NKG2D on NK-92 cells converted the immunosuppressive signal into increased cytotoxicity while preventing downregulation of NKG2D surface expression (201). This strategy has also been applied to other inhibitory receptors such as PD-1, generating a PD-1 CAR with NK-tailored endodomains such as NKG2D or DAP10/NKG2D to mediate cytotoxicity by NK cells against solid malignancies in the TME (202, 203).

### 4.5 Enhancing Homing to Navigate the Tumor Microenvironment

In addition to improving effector function within a hostile immunosuppressive environment, NK cell trafficking and retention within tumor sites is essential for optimal anti-tumor efficacy. Inducing expression of CCR7 on NK cells enhanced migration and homing to the lymph node-associated chemokine CCL19 for CD16 and rituximab-mediated ADCC against hematological malignancies (78, 204). Augmenting CAR-NK cells targeting the glioma antigen epidermal growth factor variant III (EGFRvIII) with CXCR4 expression conferred enhanced chemotaxis to U87-MG glioblastoma cells that secrete CXCL12/SDF-1α, a CXC chemokine that binds to receptors CXCR4 and CXCR7 (205). Furthermore, inducing expression of CXCR1 in NK cells with a NKG2D CAR were shown to significantly increase anti-tumor responses in subcutaneous and intraperitoneal xenograft models along with an intravenous injection model against established peritoneal ovarian cancer xenografts (206).

### 4.6 Stealth Approaches to Avoid Elimination by Host Immune Responses

#### 4.6.1 Host-Versus-Graft Immunity: A Barrier for Sustained Therapeutic Activity of Allogeneic NK Cells

NK cells have an advantage over T cells for allogeneic cell therapies because they bypass the risk of GvHD driven by αβ T-cell receptors. However, host-versus-graft (HvG) immune rejection by recipient immune cells and antibodies could limit the expansion and/or persistence of allogeneic NK cells, thus impairing their efficacy. Allogeneic HvG is the rejection of non-self donor cells due to genetic polymorphisms between the donor and recipient. In humans, this response is primarily directed against polymorphic HLA genes (major mismatch), polymorphisms in non-HLA proteins leading to “non-self” peptides presented on shared HLA alleles (minor mismatch), and red blood cell antigens (such as ABO and Rh antigens). Since NK cells do not express red blood cell antigens (207), strategies to avoid HvG for NK cell therapies are focused on avoiding rejection due to major and minor mismatch mechanisms, which are driven by CD8+ T cells, CD4+ T cells, antibodies, and to a lesser extent NK cells (208, 209).

Decades of experience in allogeneic transplantation have shown that immune rejection can occur rapidly after cell or organ transplantation, sometimes within hours if patients have pre-existing antibodies against donor HLA types (210). Allogeneic, haplo-identical (50% HLA-matched) NK cells administered to patients pre-treated with lymphodepletion usually do not persist over a month, sometimes even being eliminated within several days in lower intensity lymphodepletion regimens (74, 75, 211–213). The loss of transferred NK cells tends to coincide with the return of patient lymphocytes to baseline levels, and one study has confirmed *de novo* generation of an anti-donor T-cell response 9–14 days after NK cell transfer (75). Occasionally allogeneic donor NK cells can engraft and persist long-term in patients, which may be related to the enhanced levels of IL-15 after intense lymphodepletion (74), or the transgenic inclusion of IL-15 in the donor NK cells (110). However, another study showed that systemic IL-15, intended to support donor NK expansion, actually accelerated their loss by stimulating patient CD8+ T cells that may have eliminated the transferred NK cells (212). One approach to mitigate HvG rejection is to further deplete the immune system of the patient, for instance by adding anti-CD52 antibodies to the lymphodepletion regimen. Because CD52 is expressed in all lymphocytes (including NK cells), an engineering strategy to remove CD52 in the transferred cell therapy is required. This method has been used for allogeneic CAR-T cells and could conceivably be extended to allogeneic NK cells, but the deep and sustained depletion of the patient’s immune system can lead to severe toxicities (214). Other researchers have proposed suppressing the HvG response by engineering the cell therapy product with a 4-1BB-targeting CAR that can eliminate activated recipient lymphocytes (215). This alloimmune defense receptor (ADR) has the potential benefits of not requiring additional lymphodepleting agents and enhancing co-stimulation of the transferred cells; however, the safety and feasibility of targeting 4-1BB+ endogenous cells in patients is still unknown.

#### 4.6.2 Stealth Engineering: Evasion of Patient CD8+ T Cells and NK Cells

Many investigators have instead focused on engineering NK cells to be immunologically silent and evade the HvG response, sometimes termed “stealth.” HLA class I contains the polymorphic HLA-A, HLA-B, and HLA-C surface proteins. These molecules are heterodimers that consist of two polypeptide chains: the polymorphic HLA-encoded α chain and β2-microglobulin (β2M). HLA class I molecules are expressed in all nucleate cells and are the anchors to present intracellular peptides to CD8+ T cells (216). If the NK cell therapy is from a donor that does not share all the HLA-A, B, and C alleles of the patient, the patient’s CD8+ T cells will recognize the donor’s HLA molecules as foreign and mount a rejection response (major mismatch). Even if the donor and patient are perfectly HLA matched, patient CD8+ T cells can respond to non-self-peptides presented on the shared HLA class I molecules (minor mismatch). Thus, one mechanism to avoid both major and minor CD8+ T cell HvG is to delete or silence β2M because it is required for the surface expression of all HLA class I molecules. It has been shown that β2M-knockout (KO) NK cells do not induce an allogeneic CD8+ T cell response (217), a finding which has been shown with other cell types, including iPSCs and their derivatives (218, 219).

However, targeting β2M expression creates a problem because HLA class I molecules have an important function as inhibitory ligands for NK cells (220). HLA-C and certain HLA-A and HLA-B alleles are ligands for KIRs. HLA-E, a non-polymorphic HLA class I molecule expressed on all healthy cells, is a ligand for the NKG2A/CD94 receptor. HLA class I interactions with KIRs and NKG2A/CD94 play a major role in self-tolerance of NK cells, such that when these interactions are lost, the balance between activating and inhibitory signals on NK cells is shifted towards activation, resulting in “missing-self” lysis of target cells (26). In addition to potentially making a cell therapy product susceptible to patient NK cells, HLA class I reduction in an NK cell therapy could induce fratricide that limits the expansion and/or survival of the modified NK cells.

One strategy to solve this problem is to force expression of molecules that can inhibit NK cells, such as a transgenic HLA-E-β2M fusion protein (217). Not all NK cells express NKG2A, so other investigators have combined the HLA-E-β2M evasion strategy with genetic deletion of CD155, a ligand for the activating receptor DNAM-1, which further reduces the number of patient NK cells that are triggered by β2M-KO cell therapies (219). This finding was described with iPSC-derived T cells, so whether CD155-KO would also be relevant for NK cell therapies is an open question. Overexpression of HLA-G has also been proposed as a strategy to limit patient NK cell activation, which may also help suppress effector CD8+ T cell responses (221). Another strategy proposed to prevent NK missing-self responses of HLA class I deficient cells is to overexpress CD47 (222). CD47 is a transmembrane protein with a well-described role as a “don’t eat me” signal due to its binding to signal regulatory protein α (SIRPα) on myeloid cells (223) and high CD47 expression on tumor cells is thought to protect tumor cells from immune responses (224). Recently it was found that IL-2 stimulated NK cells upregulate SIRPα and can be inhibited through high levels of CD47 expression on β2M-KO target cells (225). Altogether, the concept to express ligands for inhibitory receptors is a promising strategy to evade patient NK cells but requires careful evaluation to ensure that trans-inhibition does not limit the function of the NK cell therapy product.

An alternative approach to avoid CD8+ T cell HvG while minimizing the induction of NK “missing-self” is to specifically delete the HLA-A, HLA-B, and HLA-C genes while leaving β2M and HLA-E intact to engage NKG2A on patient NK cells (218). This strategy still results in a loss of inhibitory KIR signaling, so it could be enhanced with additional immunosuppressive molecules like PD-L1 and CD47. In another modification of this approach, only HLA-A and HLA-B are deleted, thus providing HLA-C-driven KIR signaling in addition to HLA-E-driven NKG2A signaling (226). Although this approach opens the door to allogeneic CD8+ T cell responses to HLA-C, 12 separate banks could be made that each retain a common HLA-C allele to allow matching with >90% of patients.

#### 4.6.3 Stealth Engineering: Evasion of Patient CD4+ T Cells and Myeloid Cells

Activated NK cells express HLA class II, which, like HLA class I, is also highly polymorphic. During an allogeneic encounter, CD4+ T cells become activated through major or minor mismatch with HLA class II, leading to both enhancement of allo-reactive CD8+ T cells and direct cytotoxicity by CD4+ T cells. Additional genome editing of NK cell therapies could include the disruption of the MHC II trans-activator (CIITA) gene, a required component for HLA class II gene transcription. Several research groups have described using CRISPR technology to generate CIITA-KO hypoimmunogenic iPSC lines, either alone or in combination with β2M-KO (218, 219, 225, 227). Importantly, a cell therapy deficient for both HLA class I and II (e.g., β2M KO plus CIITA KO) will completely avoid host CD8+ and CD4+ T cell responses, in addition to evading anti-donor HLA antibodies that may exist or be generated in the patient.

Myeloid cells are crucial members of the innate immune system, where they are the first responders against infection. However, much is still unknown about the full contribution of these cells during an allogeneic response in humans; for instance, do they specifically respond to allogeneic non-self, or do they simply facilitate host T and B cell responses to allogeneic cells? A study done by Dai et al. showed that naive myeloid cells, specifically monocytes and macrophages, retain innate immune memory after the first encounter with allogeneic cells (228). This specific memory was acquired by the binding of the paired Ig-like receptor-A (PIR-A) on murine monocytes and macrophages with the MHC class I molecule on allogeneic cells. The human ortholog of PIR-A is the leukocyte IgG-like receptor (LILR) family, which contains 11 functional genes that encode five activating (LILRA1, 2, 4–6) receptors, five inhibitory (LILRB1-5) receptors, and one soluble protein (LILRA3) (229, 230). Barkal et al. showed that binding of MHC class I with the inhibitory receptor LILRB1 suppresses macrophage phagocytic activity (231). Given that some LILR genes are highly polymorphic (232), an intriguing possibility is that human myeloid cells may respond to allogeneic cells more vigorously than autologous cells and potentially acquire memory capabilities. Whether this mechanism could limit the persistence of allogeneic NK cell therapies is unknown, but one method proposed to prevent myeloid cell phagocytosis is the overexpression of CD47 (225).

Overall, there are many proposed strategies to avoid the HvG response, and their efficacy will need to be determined in clinical trials. Importantly, better characterization of patient immune responses against administered allogeneic NK cell therapies will facilitate improved stealth approaches in the future.

## 5 Conclusion

NK cells have numerous features that make them a promising cell therapy strategy for the treatment of cancer. Many trials using adoptively transferred allogeneic NK cells have demonstrated their favorable safety profile, so the main challenge for NK cell therapies is to enhance their efficacy to the level expected from CAR-T cells. A seminal study has demonstrated that allogeneic NK cells engineered to express a CAR and IL-15 have encouraging anti-tumor activity in lymphoid malignancies (110), but obstacles still remain for scalability, activity in solid tumors, and reliable persistence. Engineered iNK cells offer a highly scalable, off-the-shelf cell therapy. Modifying iNK cells with cytokine signaling and immune-evasion modules will boost their expansion and persistence, further improving clinical benefit from these therapies. Because NK cells integrate multiple receptor–ligand interactions to recognize and destroy target cells, they are naturally suited to limit the chance of tumor antigen escape, synergize with antibody therapeutics, and incorporate logic gates that can fine-tune the specificity of their response. Rapid advances in single-cell sequencing and CRISPR screening promise to deepen knowledge of NK cell signaling networks, enabling future improvements in NK cell therapies that build upon the advantageous biology of NK cells.

## Author Contributions

MA and UM-N contributed equally to this work. FR, MA, and UM-N wrote the manuscript. NF created the illustrations. All authors contributed to the article and approved the submitted version.

## Conflict of Interest

FR, NF, AS, MA, and UM-N are current employees at Janssen Research and Development, LLC, Pharmaceutical Companies of Johnson & Johnson.

## Publisher’s Note

All claims expressed in this article are solely those of the authors and do not necessarily represent those of their affiliated organizations, or those of the publisher, the editors and the reviewers. Any product that may be evaluated in this article, or claim that may be made by its manufacturer, is not guaranteed or endorsed by the publisher.
